# Mooren’s ulcer: a multifactorial autoimmune peripheral ulcerative keratitis and current treatment protocols

**DOI:** 10.3389/fmed.2025.1630585

**Published:** 2025-11-11

**Authors:** Shangkun Ou, Yujie Zhang, Yuchong Feng, Xueer Zheng, Yuan Lin, Liying Zhang, Su Zhao, Yu Su, Han Cai, Longqin Lin, Hao Gu, Huping Wu, Yiming Wu

**Affiliations:** 1 Department of Ophthalmology, The Affiliated Hospital of Guizhou Medical University, Guiyang, China; 2 Xiamen Eye Center and Eye Institute of Xiamen University, School of Medicine, Xiamen, China; 3 Department of Ophthalmology, The Qinglong County People's Hospital, Qinglong, China; 4 Fujian Provincial Key Laboratory of Ophthalmology and Visual Science, Fujian Engineering and Research Center of Eye Regenerative Medicine, School of Medicine, Xiamen University, Xiamen, China; 5 Department of Biomedical Sciences, School of Infection, Inflammation and Immunology, College of Medicine and Health, University of Birmingham, Birmingham, United Kingdom

**Keywords:** autoimmune corneal diseases, corneal melt, filarial nematodes, hepatitis C, HLA, hookworm, Mooren’s ulcer, peripheral ulcerative keratitis

## Abstract

Mooren’s Ulcer (MU) is a rare, chronic, and painful form of autoimmune peripheral ulcerative keratitis (PUK), with an elusive etiology and a risk of corneal perforation and vision loss. Despite numerous proposed triggers, including parasitic infections, hepatitis C virus, ocular trauma, and surgery, the pathogenesis of MU remains unclear, and diagnosis continues to rely heavily on exclusion. A key controversy in current clinical practice lies in the absence of standardized diagnostic criteria and consensus treatment protocols. This review addresses this gap by presenting a comprehensive and structured diagnostic framework for MU, particularly emphasizing laboratory and immunological testing strategies to facilitate accurate differential diagnosis. To our knowledge, this is the first review to systematize these diagnostic components in detail. In addition to summarizing the latest findings on epidemiology, etiology, pathology, and classification, the work also review the evolving role of advanced imaging, histopathology, and tear-based markers in MU diagnosis and monitoring. Treatment options, ranging from immunosuppressive therapy to surgical intervention, are discussed based on disease severity. This work recommend a tiered, individualized approach to treatment and advocate for future multicenter studies to validate diagnostic protocols and establish evidence-based clinical guidelines.

## 1 Introduction

Mooren’s ulcer (MU) is a significant subset of autoimmune peripheral ulcerative keratitis (PUK), accounting for approximately 35% of cases (1). As with other forms of PUK, MU is characterized by a progressive inflammatory process affecting the juxtalimbal cornea, resulting in crescent-shaped stromal thinning, epithelial defects, and infiltration of inflammatory cells within the corneal stroma (2, 3). First proposed by Bowman in 1849 and later described in greater detail by Mooren in 1867, MU has since been recognized as a chronic, idiopathic, progressive, and painful variant of PUK (3, 4). Typically, MU presents as a grey, swollen patch located about 2–3 mm from the limbus. This lesion rapidly progresses, forming a furrow that moves toward the central cornea, potentially involving the entire corneal surface while usually sparing the sclera (5, 6). The ongoing progression of these ulcers can lead to corneal perforation. Notably, approximately one-third of MU patients exhibit bilateral involvement.

MU is believed to involve genetic and autoimmune components, but its exact pathogenesis remains unknown. Several factors have been implicated in its development, including intestinal parasite infections (7), chronic hepatitis C infection (8), ocular trauma (9), surgical interventions (10), and pterygium, particularly following pterygium surgery at the donor site after conjunctival limbal graft (11). Despite the use of various therapeutic strategies, including local and systemic immunosuppressants, cytotoxic drugs, anti-inflammatory agents, and surgical treatment such as conjunctival resection and keratoplasty, there is no established standardized treatment protocol for MU. Therefore, this review delves into the intricate details of epidemiology, etiology, risk factors, pathology, clinical manifestations and classification, diagnosis, differential diagnosis, investigational techniques, treatment, efficacy evaluation, and prognosis, aiming to bridge the existing gaps and controversy, especially in full diagnostic techniques and different options of stepladder approach, in the management of MU patients and provide a clearer guidelines for clinicians and researchers in their efforts to understand and treat this complex condition.

## 2 Epidemiology

MU occurs infrequently in the northern hemisphere, whereas it is more prevalent in regions such as the southern hemisphere, the Indian subcontinent, China, and central Africa (12–14). The incidence, clinical characteristics, and severity of MU vary significantly across geographical regions and among different racial groups (15). Chen et al. (16) analyzed 550 cases (715 eyes) of MU treated in China from 1960 to 1996. The study reported a mean onset age of 48.4 years and a male-to-female ratio of 1:0.74, suggesting that men were 1.35 times more likely to be affected than women. In Ibadan, southwest Nigeria, a study reported a male-to-female ratio of 1:0.28, indicating a significantly higher number of male patients (17). Wood and Kaufman (18) suggest that MU has a higher prevalence in men. Consistent with this observation, Raghav et al. recently reported that males account for 90% of MU patients in rural India (19). While the exact reasons for this discrepancy are still uncertain, contributing factors may include a higher incidence of ocular injuries among men and potential biological differences.

## 3 Etiology and risk factors

**Figure 1:**
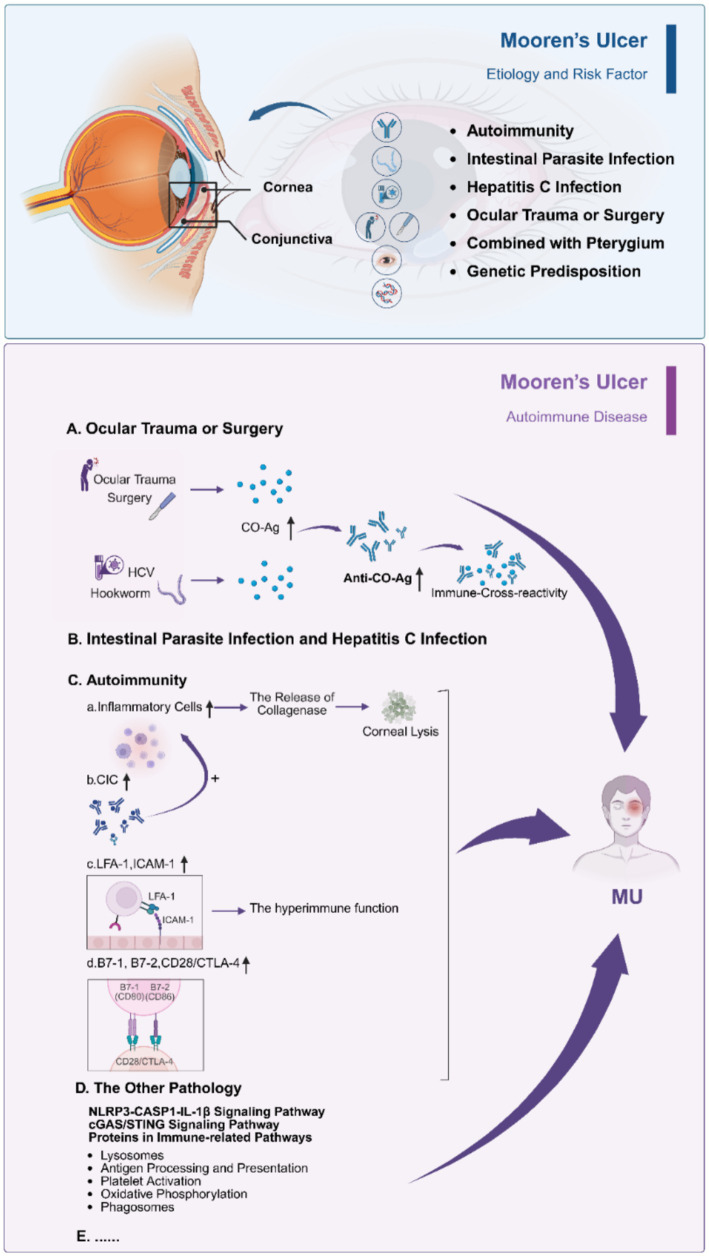
The etiology and pathology of Mooren’s ulcer. Autoimmunity, intestinal parasite infection, hepatitis C virus infection, ocular trauma or surgery, coexistence with pterygium, and genetic predisposition are recognized risk factors. Corneal injury or infection may expose the cornea-associated antigen (CO-Ag), inducing anti-CO-Ag antibodies and immune cross-reactivity. Activated inflammatory cells and circulating immune complexes (CICs) promote collagenase release and corneal lysis, while adhesion molecules (LFA-1, ICAM-1) and costimulatory pathways (B7/CD28/CTLA-4) sustain local hyperimmunity. Upregulation of innate immune cascades, including NLRP3-CASP1-IL-1β and cGAS/STING signaling, further contributes to progressive stromal destruction. Created in BioRender. Wu, Y. (2025) https://BioRender.com/xn992hu.

MU is associated with a wide range of possible causes and risk factors (Figure 1), most of which fall into the categories outlined below:

### 3.1 Autoimmunity

Accumulating evidence suggests that MU is associated with immune dysfunction (13, 20, 21). The main evidence for this inference includes:

(a) Serum analyses of MU patients have revealed the presence of cornea-associated antigen (CO-Ag) and circulating autoantibodies targeting corneal tissue (22–25). In addition, indirect immunofluorescence testing revealed that 75% of MU patients had serum antibodies against rabbit corneal epithelial cells, and 37.5% had antibodies against human corneal epithelial cells, further supporting the presence of autoantibodies in MU patients (26).
(b) MU patients’ serum exhibits elevated levels of IgG and IgA (27).
(c) Immunological analyses of peripheral blood have shown an elevated helper-to-suppressor T cell (Th/Ts) ratio, accompanied by a reduction in suppressor T cells (Ts) (28, 29). Consistently, another study reported significantly reduced OKT8 + T cells and elevated OKT4+/OKT8 + ratios in MU patients compared to controls, suggesting regulatory imbalance in cellular immunity (26).
(d) Abnormally high levels of circulating immune complexes (CICs) have been observed in the serum (29).
(e) High expression of lymphocyte function-associated antigen-1 (LFA-1) and intercellular adhesion molecule-1 (ICAM-1) has been observed in the bulbar conjunctival tissue adjacent to the ulcer, along with significant infiltration of polymorphonuclear granulocytes, plasma cells, and lymphocytes (30).
(f) Additionally, immunoreactive cells and cytokines are present at the corneal limbus, with complement 1 (C1) more concentrated in the periphery than at the center of the cornea. Antigen–antibody complexes within the corneal limbal vessels can activate C1, initiating a classical complement cascade reaction (31, 32).
(g) The fact that the erosive corneal ulcer originates at the corneal limbus further supports the theory that it is an immune-related disease (21).

### 3.2 Intestinal parasite infection

Intestinal parasite infection is a risk factor for inducing MU. Zelefsky et al. identified a link between hookworm infection and the development of MU, especially among elderly men (7). The amino acid sequence encoded by the cDNA of CO-Ag, a stromal protein possibly involved in MU pathogenesis, was shown by Gottsch et al. to be identical to calgranulin C, a neutrophil protein present on filarial nematodes (23, 33). So, they considered that calgranulin C on the surface of parasites after parasite infection, such as filarial nematodes and hookworm, stimulates the body to produce cross immune response to cornea, resulting in the occurrence of MU (7, 23, 33). However, more than 50% of patients did not show hookworm infection (7), and the correlation with hookworm infection should be further confirmed.

Although the hypothesis of molecular mimicry between corneal CO-Ag and parasite-derived calgranulin C is intriguing, current evidence remains limited to case reports and small, uncontrolled series from endemic areas. Over half of reported MU patients had no confirmed parasitic infection, and diagnostic methods for helminth detection varied widely. Therefore, the existing data suggests a possible association rather than a proven causal link. Larger, controlled studies using standardized parasitological testing are needed to clarify this relationship.

### 3.3 Hepatitis C infection

The relationship between hepatitis C virus (HCV) infection and MU is controversial. Successful treatment of HCV infection with ribavirin and interferon therapy has been associated with remission of corneal symptoms in MU patients (34). However, some research considered that effect may be related to the immunomodulatory effects of interferon (35). What’s more, Pluznik et al. found that corneal symptoms worsened with anti-HCV treatment for chronic HCV infection in MU patients (36). Furthermore, Wang et al. performed serological screening for HCV infection on eight MU patients and found that all patients tested negative for HCV serology (37). Although the precise link between HCV infection and MU pathogenesis remains unclear, MU patients with HCV infection should be carefully managed.

Reports describing improvement of MU after antiviral therapy and others showing disease exacerbation during interferon treatment indicate that the observed effects may reflect immunomodulatory mechanisms rather than a direct viral association. In addition, several cohort studies failed to detect HCV infection in MU patients. Taken together, the evidence remains inconsistent and of low certainty. Current evidence suggests that HCV infection could be an associated comorbidity, although a direct causal role has not yet been established.

### 3.4 Ocular trauma or surgery

Ocular trauma or surgery is increasingly recognized as a contributing factor to MU. According to Chen et al., 10.7% of MU cases (550 patients, 715 eyes) in China were preceded by surgical procedures or ocular trauma (16). Additionally, Kim et al. reported that 41.7% (10/24) of patients had prior infection, trauma, or ocular surgery (15). These findings collectively imply a potential role of corneal injury in the development of MU. Given the current lack of uniform diagnostic standards for MU, clinicians should exercise caution when assessing corneal ulcers post-trauma or surgery. Furthermore, the mechanism through which ocular trauma contributes to MU remains unclear. In a case reported by Toyokawa et al., bilateral MU developed after EX-PRESS glaucoma implantation, implicating a possible autoimmune mechanism affecting the peripheral cornea (38). Surgical procedures or ocular traumas to the cornea disrupt its local structures, exposing normally hidden collagen type I, CO-Ag, thus triggering an autoimmune response and heightening the likelihood of MU development (7, 14). Further investigation into these aspects is warranted.

### 3.5 Combined with pterygium

Pterygium is a common, benign growth of conjunctival tissue that extends in a wedge-shaped form onto the cornea (39, 40). Ulcerations can occur at the donor site following pterygium excision (11). Several cases of MU occurring in conjunction with pterygium have been reported (41, 42). Pterygium could impair the integrity of the cornea and lead to the exposure of CO-Ag, potentially triggering MU (42). Although the exact mechanism is unclear, the presence of corneal ulceration in conjunction with pterygium should raise suspicion for MU.

### 3.6 Genetic predisposition

Through molecular biological methods, the cause of corneal ulcers, such as MU, can be located to certain genetic changes (43). In recent years, several researchers have reported a strong correlation between MU and the expression of ocular surface inflammation marker, human leukocyte antigen (HLA) (43). Taylor et al. reported elevated frequencies of HLA-DR17 and HLA-DQ2 alleles, with statistically significant differences [3]. Zelefsky et al. later found that HLA-DR17 was more strongly associated with MU than HLA-DQ2 (44). Additionally, Liang et al. found that HLA-DQ5 was also associated with MU (45). However, the mechanism by which HLA genotypes confer susceptibility to MU remains unclear and warrants further investigation through genetic studies involving diverse populations and larger sample sizes.

### 3.7 Other potential immunogens

Some reports suggest that external immunogens, such as vaccines, can potentially induce corneal diseases through immune-mediated mechanisms (46). Consequently, vaccines may also influence the occurrence or progression of MU. In a Moroccan case reported by Alliti et al., MU developed in a patient with prolonged thalidomide exposure shortly after an inactivated COVID-19 vaccination. Vision loss occurred a week after the second dose, pointing to a possible immune-mediated link (47). However, isolated case reports do not necessarily establish a definitive clinical link between vaccines and MU. The patient may have had other comorbidities or underlying health and immunological conditions that were not thoroughly documented prior to vaccination (48).

## 4 Pathology

While the underlying mechanisms of MU remain elusive, it is generally recognized as an autoimmune disease involving both cellular and humoral pathways (Figure 1). The cornea and conjunctiva near ulcer sites have been found to contain CO-Ag (33), HLA class II molecules, and infiltrating inflammatory cells, indicating localized immune activation (14, 44, 45). The conjunctiva and its associated vasculature also appear to contribute to corneal destruction (49).

CO-Ag, localized within the corneal stroma and sharing sequence identity with human neutrophil calgranulin C (23, 33), is regarded as a potential key autoantigen in MU. High serum levels of anti-CO-Ag autoantibodies have been reported in MU patients (24). Immune responses to CO-Ag may be triggered by corneal trauma or surgery, or through molecular mimicry with pathogens such as HCV or intestinal parasites, thereby initiating or amplifying autoimmune injury. Although the precise biological role of CO-Ag remains uncertain, it likely serves as an immunogenic stimulus that sustains chronic inflammation. Elucidating its properties may help identify disease activity early and inform targeted therapies.

Histopathologic studies have demonstrated marked infiltration of T lymphocytes, together with smaller numbers of B cells, macrophages, neutrophils, NK cells, and mast cells (20, 28). Hyperactive immune responses in the peripheral cornea and adjacent bulbar conjunctiva are mediated through adhesion molecules such as ICAM-1 and LFA-1. The colocalization of CD28/CTLA-4 with B7-1 and B7-2 beneath the epithelium suggests further immune-regulatory interactions (13). Elevated circulating immune complexes may enhance inflammatory cell recruitment and collagenase release, leading to stromal lysis and progressive ulceration (50).

Recent molecular studies have expanded understanding of MU pathogenesis. Li et al. reported upregulation of the NLRP3-CASP1-IL-1β pathway in conjunctival tissue (51). Hao et al. generated a proteome atlas showing enrichment of immune-related pathways, including lysosomal activity, antigen presentation, platelet activation, oxidative phosphorylation, and phagosomes (52). Zhang et al. found increased expression of cGAS/STING signaling components in corneal epithelial cells of MU patients (53). These findings highlight multiple innate immune cascades that may converge to drive corneal inflammation, although their precise contributions require further clarification.

## 5 Clinical manifestations and classification

**Figure 2:**
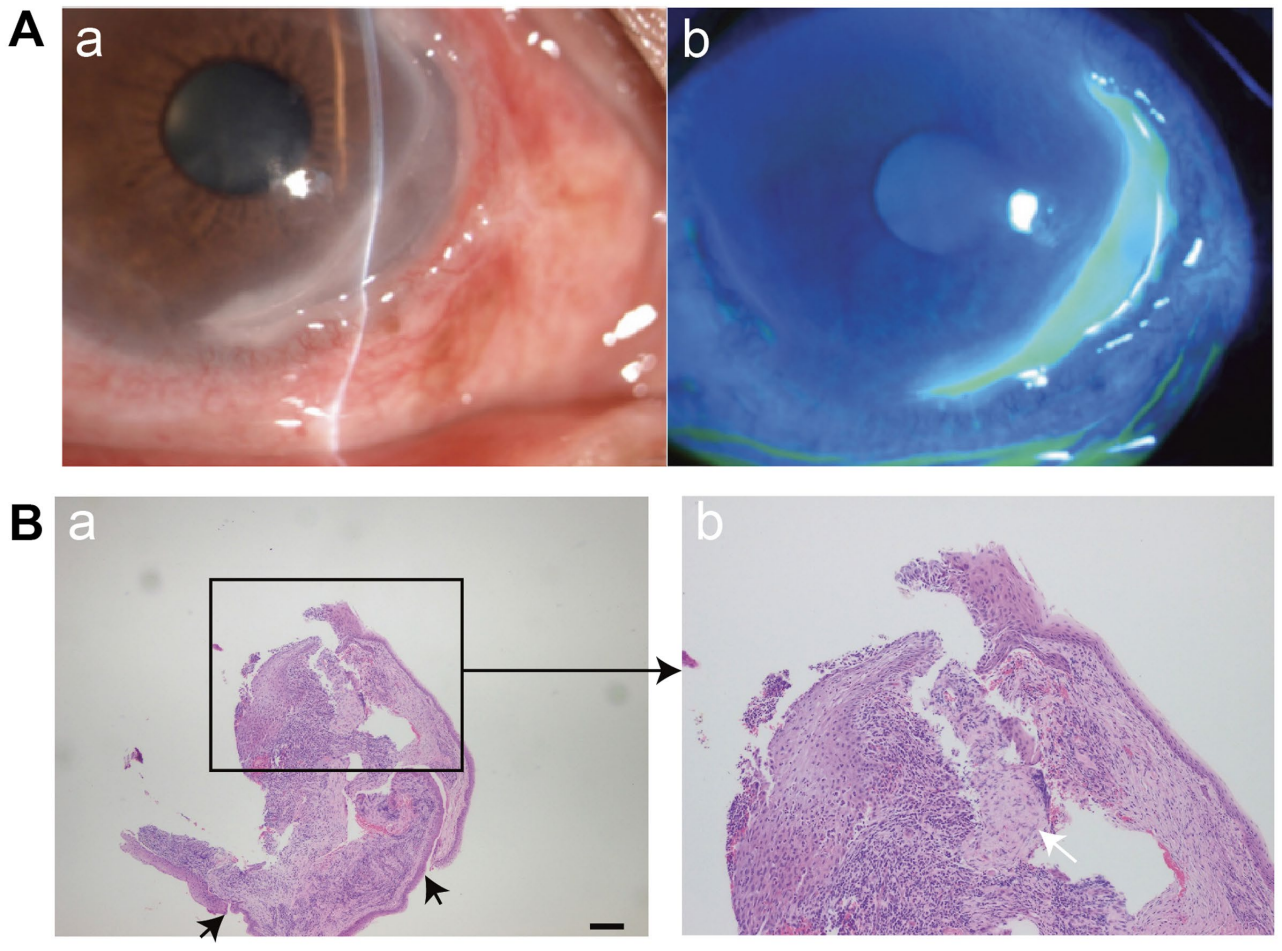
**(A) (a,b)** Slit-lamp microscopy demonstrates the typical morphology of MU, including steeply overhanging central and leading edges, and a thin, vascularized ulcer base. The silt lamp image was reproduced and permitted from Ou S, et al (copyright © 2024 The Authors. Published by Elsevier Ltd. CC BY-NC 4.0) (54). **(B)** Histopathological features of MU. **(a)** Typical findings include epithelial detachment and loss of the anterior elastic membrane (black arrows). Scale bar = 200 μm. **(b)** Dense infiltration of inflammatory cells (white arrow) and stromal degeneration are evident in the corneal tissue. The image was reproduced and permitted from Zhang et al. (56) [copyright © 2022 The Author(s). Published by Informa UK Limited, trading as Taylor & Francis Group. CC BY-NC-ND 4.0].

MU typically presents with severe ocular pain, marked redness, photophobia, and excessive tearing. Typical features include intense limbal inflammation and ulceration of the corneal rim with a disrupted advancing edge containing many blood vessels [Figures 2A (a,b)] (54). In most cases, stromal involvement ranges from one-third to two-thirds of the corneal thickness, and the sclera remains unaffected. Studies have shown that 45% of corneal lesions involve half of the limbus, 21% extend to the entire limbus, and 70.1% involve the eyelid fissure (16). It may relate to eyelid fissure exposure and a relative deficiency of limbal stem cells in the affected area.

Wood and Kaufman (18) proposed a two-subtype classification of MU based on factors such as prognosis, clinical presentation, and age at onset. Type 1, considered the benign variant, typically affects only one eye, presents with relatively mild symptoms, and occurs in individuals over 35 years old. Patients in this group generally respond well to both pharmacological and surgical interventions. Type 2, in contrast, represents the more aggressive form. It often involves both eyes, is associated with severe symptoms, and is seen predominantly in patients under the age of 35. This type is typically resistant to conventional treatment approaches.

Currently, the commonly used classification, according to clinical images Watson (12) publication, based on clinical manifestations and low-dose anterior segment fluorescein angiography, divides MU into three types (Table 1) (3):

(a) Unilateral Mooren’s ulceration (UM): UM is rare and typically occurs in patients over 60 years old, characterized by the rapid onset of redness and severe pain in one eye. UM is characterized by poor response to analgesics, intense inflammation, conjunctival congestion, but no scleral inflammation or necrosis. Anterior segment fluorescein angiography found occluded conjunctival vessels and local episcleral venular, along with structural disruption and vascular leakage at the limbus and ulcer base.
(b) Bilateral aggressive Mooren’s ulceration (BAM): Commonly observed in the Indian subcontinent and East Africa, BAM usually affects patients from 14 to 40 years of age and presents with bilateral disease accompanied by mild eye pain. When one eye is diagnosed, the other eye may not have obvious symptoms, even though the conjunctiva is congested. If left untreated, the unaffected eye will develop a gray patch within the corneal stroma, followed by a gray patch forming along the limbus edge approximately 2 mm from the edge. Over a few days, these gray spots coalesce and tissue loss occurs, resulting in the typical appearance. Unlike UM, ulcers in BAM can perforate spontaneously. Angiography reveals closure of episcleral vessels in some areas.
(c) Bilateral indolent Mooren’s ulceration (BIM): It is more common in middle-aged patients with discomfort in both eyes. Ulcers in both eyes typically occur almost simultaneously or within a few days of each other, although usually, one eye exhibits a more severe reaction. Many ulcers will heal spontaneously. Angiography shows no detectable changes in episcleral or conjunctival vessels, but there is an abnormality in the limbal circulation, with new blood vessels extending to the base of the ulcer.

**Table 1:** Watson’s classification of MU types.

Features	BIM	BAM	UM
Affected Sides	Involves both sides	Typically affects both sides	Generally one-sided
Gender	No strong gender association	More frequent in males than females	More frequent in females than males
Age	Primarily middle-aged to elderly	Common in younger individuals	Mostly found in older adults
Progression	Slow	Slow, aggressive at presentation	Rapid
Pain Severity	Mild discomfort	Moderate to severe pain	Severe pain
Recurrence Risk	Rare	Common	Common
Complications	Rare	Perforation	Rare

## 6 Diagnosis

MU is primarily diagnosed through a process of exclusion (Table 2 introduces normal ocular examination, imaging examination, and laboratory test for MU in previous publications; Table 3 introduces potential autoimmune markers for excluding other diseases) (9, 10, 35, 38, 41–43, 49, 55–65). Any disease capable of causing PUK must first be excluded (α1-antitrypsin deficiency, Beçhet’s disease, malignancy, infections (AIDS, bacillary dysentery, borreliosis, gonorrhea, hepatitis C, herpes zoster, syphilis, tuberculosis), inflammatory bowel disease, polyarteritis nodosa, progressive systemic sclerosis, systemic lupus erythematosus, sjögren syndrome, scleroderma, sarcoidosis, rheumatoid arthritis, relapsing polychondritis, and Wegener granulomatosis) (4, 35, 50), and the diagnosis of MU relies on a detailed medical history and typical ulcer morphology.

**Table 2:** MU diagnostic workup: examination types, procedures, and clinical rationale.

Type of examination	Specific test/Procedure	Purpose and potential findings
Ocular Examination	Slit-lamp biomicroscopy	To identify peripheral corneal ulceration, epithelial defects, and characteristic overhanging edges of Mooren’s ulcer. Helps rule out infectious keratitis or trauma-related ulcers.
	AS-OCT (Anterior segment optical coherence tomography)	Provides detailed corneal structure visualization, including stromal thinning and depth of ulceration. Supports diagnosis and tracks healing. Helps rule out deeper infectious or structural pathologies.
	Fundus examination	To assess posterior segment and exclude other intraocular pathologies.
Imaging	Chest X-ray	To rule out systemic diseases like TB or sarcoidosis.
	Ultrasound B-scan	Used when media opacity precludes fundus exam, especially in severe MU.
	Sinus and KUB radiographs	Performed to rule out other systemic inflammatory causes.
Laboratory test	Complete blood count (CBC)	Assesses systemic inflammation, anemia, infection. Helps rule out systemic causes of ulceration.
	ESR and CRP	Markers of systemic inflammation; elevated in some MU patients. Help differentiate from systemic autoimmune diseases.
	Hepatitis B and C serology	Investigates possible viral associations with MU. Some cases had positive Hep C.
	HIV serology	To assess for immunosuppression as a possible contributing factor.
	Urinalysis and stool exam	To assess for systemic infection or parasitic infestation.
	Liver and renal function tests	To evaluate for systemic health and exclude metabolic contributors.
	Serum ACE and QuantiFERON test	To rule out sarcoidosis and tuberculosis.
	Corneal scraping and culture	To exclude infectious keratitis. All cases with this test returned negative microbiology.
	Corneal or conjunctival biopsy	Histological confirmation of MU features. Helps rule out neoplastic or infectious causes.
	Autoimmune markers	To exclude autoimmune diseases which can mimic or contribute to PUK. Negative in all MU cases confirming idiopathic etiology.

**Table 3:** Autoimmune serology panel: disease exclusion in differential diagnosis of MU.

Autoimmune marker	Disease(s) ruled out
ANA (Antinuclear antibodies)	Systemic sclerosis, Sjögren’s syndrome, systemic lupus erythematosus
ANCA (Anti-neutrophil cytoplasmic antibodies)	Granulomatosis with polyangiitis, microscopic polyangiitis, IBD-related vasculitis
RF (Rheumatoid factor)	Rheumatoid arthritis
Anti-CCP (Anti-cyclic citrullinated peptide)	Rheumatoid arthritis
Anti-dsDNA (Double-stranded DNA Antibody)	Systemic lupus erythematosus
SSA/Ro (Anti-Ro antibodies)	Systemic lupus erythematosus, Sjögren’s syndrome
SSB/La (Anti-La antibodies)	Sjögren’s syndrome
Cardiolipin antibody	Antiphospholipid syndrome, systemic lupus erythematosus
Complement (C3, C4)	SLE, Immune complex-mediated diseases, Infection-related immune activation
MPO (Myeloperoxidase antibody)	Microscopic polyangiitis
PR3 (Proteinase 3 antibody)	Granulomatosis with polyangiitis
Lupus anticoagulant	Antiphospholipid syndrome
RNP (Anti-ribonucleoprotein antibody)	Mixed connective tissue disease

Infectious corneal disease can be ruled out by scraping and culture of corneal and conjunctival secretions. Comprehensive laboratory tests, including antistreptococcal antibodies, rheumatoid factor, circulating immune complexes, antinuclear antibodies erythrocyte sedimentation rate, and complete blood count, can be conducted to exclude other causes of PUK (66, 67). In recent years, histopathological examination has been increasingly utilized to aid in the diagnosis of MU through typical histopathological manifestations.

## 7 Differential diagnosis

MU is an exclusionary disease, requiring the exclusion of other diseases causing peripheral corneal ulcers through detailed clinical presentation and laboratory tests (Tables 2, 4).

**Table 4:** Differential diagnosis of MU.

Diseases	Pain	Visual loss	Epithelial defect	Location	Disease progression	Association
MU	+	+	+	Starts in the peripheral region, with progression both circumferentially and centrally; typically bilateral in the malignant form and unilateral in the limited form.	Progressive	Inflamed conjunctiva
CVD-associated PUK	+	+	+	Anywhere of the peripheral cornea, bilateral	Progressive	Constitutional symptoms and other systemic manifestations
TMD	−	±	−	Mostly affect superior cornea, and rarely involving the inferior, unilateral or bilateral	Slow	none
Fuchs’ superficial marginal keratitis	+	+	−	Irregular	Mild	Remissions and relapse
Marginal keratitis	+	+	+	Anywhere of the cornea, unilateral	Benign, microbial keratitis is rapid progress	Hypersensitivity caused by blepharoconjunctivitis associated organisms
PMD	−	±	−	Mostly affect Inferior cornea, bilateral, asymmetric	Mild	Corneal ectatic diseases
Senile marginal furrow degeneration	−	−	−	Circumferential	Mild	None
Arcus senilis	−	−	−	Circumferential, bilateral and symmetric	Mild	Hypercholesterolemia

*“+” means yes, “-” means no, “±” means not sure. CVD-associated PUK: collagen vascular diseases-associated peripheral ulcerative keratitis, MU, Mooren’s ulcer; PMD, pellucid marginal degeneration; TMD, terrien’s marginal degeneration.

### 7.1 Collagen vascular disease-associated PUK

Collagen vascular diseases (CVDs), including systemic lupus erythematosus, relapsing polychondritis, polyarteritis nodosa, Wegener’s granulomatosis, and rheumatoid arthritis, need to be considered. In CVD-associated PUK, inflammation typically extends to adjacent conjunctiva, episclera, and sclera, whereas MU generally lacks scleral involvement. Unlike MU, CVD-associated PUK is often a manifestation of systemic disease. CVD-associated PUK represents an ocular manifestation of an underlying systemic condition (68). In contrast, MU occurs in the absence of any diagnosable systemic disorder (4).

### 7.2 Terrien’s marginal degeneration

Terrien’s marginal degeneration (TMD) typically presents as bilateral thinning of the superior peripheral cornea (69, 70), progressing more slowly compared to MU. TMD presents with superficial vascularization and a distinct opaque line of fine cholesterol crystals separating it from the central cornea (71, 72). Acute painful inflammation is rare in TMD cases (73).

### 7.3 Fuchs’ superficial marginal keratitis

Fuchs’ superficial marginal keratitis manifests with recurrent episodes of ocular irritation and progressive thinning of the marginal superficial stroma, occasionally accompanied by pseudopterygium (74, 75). The progressing ulcer is bordered by a gray demarcation line separating it from the central cornea (72).

### 7.4 Marginal keratitis

Marginal keratitis is an inflammatory condition caused by various organisms, including viruses, bacteria, fungi, parasites, and chlamydiae. Bacterial corneas with peripheral involvement often display purulent infiltrates. Although corneal cultures may be negative, pathogenic organisms can sometimes be isolated from the ipsilateral lid margin or conjunctiva (76). Unlike MU, which involves a sterile inflammatory response, marginal keratitis is infectious in nature. For instance, staphylococcal marginal keratitis typically manifests as a peripheral corneal infiltrate accompanied by epithelial disruption. A distinct separation between the infiltrate and the limbus is usually observed, and the condition is commonly associated with underlying blepharitis. While patients may report symptoms such as photophobia and ocular discomfort, the intense and incapacitating pain characteristic of Mooren’s ulcer is generally absent.

### 7.5 Degenerative corneal diseases

This category includes arcus senilis, senile marginal furrow degeneration, and pellucid marginal degeneration (PMD). PMD predominantly involves the inferior peripheral cornea, presenting as crescent-shaped thinning while maintaining corneal transparency (77). Arcus senilis and senile marginal furrow degeneration are physiological changes that, unlike MU, are usually not visually significant (76).

## 8 Application of new and investigational techniques in MU

### 8.1 Three-dimensional anterior segment optical coherence tomography

Conventional corneal topography methods face challenges in analyzing corneal shape due to ulcer infiltration and corneal clouding, particularly in severe cases (78, 79). Three-dimensional anterior segment optical coherence tomography (3D AS-OCT) offers enhanced imaging depth and higher-definition cross-sectional images of the cornea (80, 81). Masahito Yoshihara et al. used 3D AS-OCT to utilize 3D AS-OCT to analyze corneal topography and visual function in MU cases, dividing the patients’ axial power maps into arcuate, crab-claw and intermediate maps. They observed decreased best-corrected visual acuity (BCVA) and increased regular and irregular astigmatism when the lesion was near the corneal center (82). Future applications of 3D AS-OCT hold promise in quantitatively assessing the impact of corneal thinning on optical quality and visual function in MU. However, it is important to note that 3D AS-OCT cannot always provide a definitive differential diagnosis of MU. For instance, both TMD and MU may present with similar imaging features, such as a central flattened zone surrounded by steeper regions and a crab-claw pattern on the corneal axial power map (82, 83).

### 8.2 *In vivo* confocal microscopy

In vivo confocal microscopy (IVCM) is a non-invasive diagnostic tool valuable for evaluating various ocular surface diseases (84, 85). Recent research efforts have explored the utility of IVCM in MU patients (86, 87). Shin Hatou et al. (87) have shown that patients with active ulcers exhibit higher inflammatory cell density (ICD) compared to those in remission. Furthermore, ICD tends to decrease over time with immunosuppressive therapy. Additionally, numerous dark cysts containing polymorphs were observed in patients with active ulcers, but not in those in remission (87). The observation of a large number of fluid-filled cysts in the IVCM is likely to suggest imminent perforation. Thus, IVCM can aid in assessing inflammation severity, treatment response, and predicting corneal perforation risk in MU patients.

### 8.3 Histopathological examination

Several studies have conducted histopathological examinations of corneal tissues from MU patients. Hematoxylin–eosin (HE) staining revealed epithelial hyperplasia in adjacent conjunctiva and inflammatory infiltration in stroma (20, 56) (Figure 2B). Immunohistochemical (IHC) staining demonstrated strong expression of inflammatory markers such as CD34, c-kit, STRO-1 cells (88), ICAM-1, LFA-1, NLRP3 (51) in MU specimens. Li et al. founded high levels of GPR91 protein were observed in conjunctival and/or corneal tissues of MU patients (89). The histopathological findings from the conjunctiva adjacent to ulcer, limbus and ulcerated area of the cornea in Table 5 (90). So the histopathological examinations revealing an increase in local inflammatory vesicles or immunohistochemical staining demonstrating high expression of inflammatory factors can be instrumental in confirming the diagnosis of MU when clinical diagnosis is inconclusive.

**Table 5:** Histopathological findings in MU.

Section		Performance
Adjacent conjunctiva	Epithelium and basement membrane	Normal
	Stroma	Hyperemia and edema
	Inflammatory infiltrations	Less numerous eosinophils, neutrophils and mast cells; Mainly by plasma cells and lymphocytes
Corneal stroma at limbus	Superficial zone	Vascularized with perivascular infiltration (mainly by lymphocytes and plasma cells of various densities)
	Central zone	Fibroblastic activity
	Deepest zone	Macrophages infiltration
Corneal ulcer	Ulcer base	Necrobiotic material and inflammatory cell infiltration
	Central edge of the ulcer	Thickened stroma, absence of inflammatory infiltration
	Peripheral edge of the ulcer	Dense inflammatory infiltration including plasma cells, lymphocytes, neutrophils, histiocytes and mast cells

### 8.4 Ocular surface microflora testing

The ocular surface microbiota establishes a unique microecology on the ocular surface, closely intertwined with human immune defense mechanisms and the development of ocular diseases (91, 92). The innate immune activity of the ocular surface epithelium, as highlighted by Ueta et al. (93), contributes to establishing symbiotic interactions with commensal bacteria. In recent years, advancements in 16S rDNA sequencing technology have facilitated the detection of ocular surface microbiota (94). Presently, 16S rDNA sequencing can distinguish bacterial flora variations in patients with conditions like infectious keratitis, dry eye, and conjunctivitis. However, research on the ocular surface bacterial flora in immune-related keratitis, including MU, remains scarce. Thus, investigating the ocular surface microecology in MU ulcer patients holds promise for deeper insights into the disease.

## 9 Treatment

**Figure 3:**
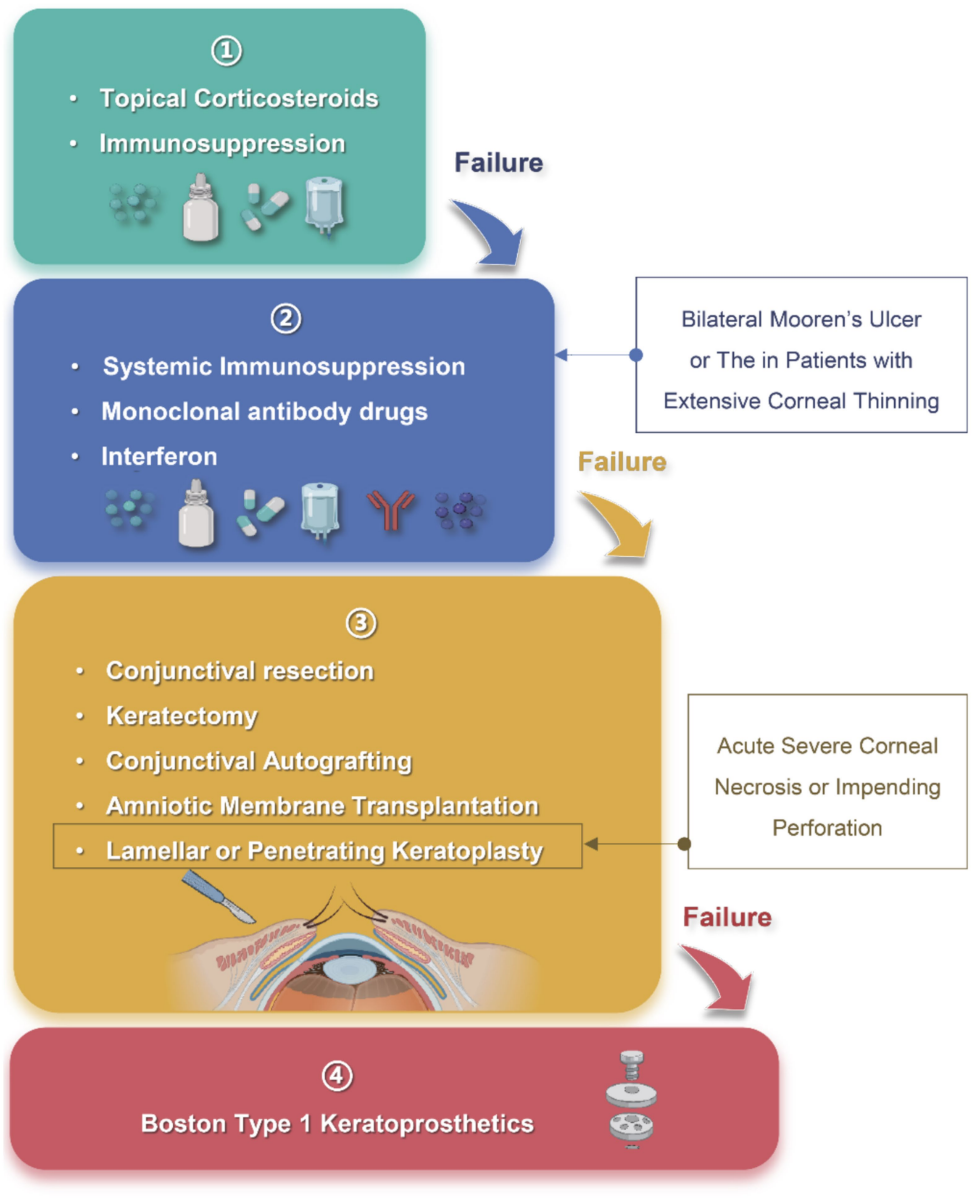
“The stepladder approach” in the treatment of Mooren’s ulcer. Step 1: Topical corticosteroids and immunosuppressants are used as first-line therapy for mild, unilateral disease; Step 2: Systemic immunosuppression, monoclonal antibodies and interferon are indicated for bilateral or refractory cases with progressive corneal thinning; Step 3: Surgical procedures, including conjunctival resection, keratectomy, conjunctival autografting, amniotic membrane transplantation, or lamellar/penetrating keratoplasty, are considered when medical therapy fails or when acute corneal necrosis or impending perforation develops; Step 4: Boston type 1 keratoprosthesis serves as a last-resort option after repeated graft failure or end-stage corneal destruction. Arrows indicate escalation to the next level of intervention when disease control is inadequate. Created in BioRender. Wu, Y. (2025) https://BioRender.com/x9geu1w.

Some researchers advocate for a “stepladder approach (55, 95)” in treating MU (Figure 3), tailoring interventions based on the severity of the disorder. This approach typically involves a combination of medication [both topical and systemic, Table 6 (95–101)] and surgical therapy. Among them, due to the irreversible destruction of the corneal anatomy by surgical therapy, surgical therapy is often carried out when the medication can not control the MU symptoms.

**Table 6:** Classic treatment strategies and follow-up protocols for different clinical presentations of MU.

Clinical features	Treatment modality	Dosage and administration	Follow-up protocol
Unilateral involvement, <2 quadrants of peripheral corneal damage, <50% stromal thinning	Topical corticosteroids	Dexamethasone eye drops (1.5 mg/mL), 6 times daily	Every 3 days during acute phase until healing; every 3 months for 6 months; thereafter as needed
Bilateral involvement, >2 quadrants of peripheral corneal damage, >50% stromal thinning	Systemic corticosteroids	Oral prednisolone 1–1.5 mg/kg/day	Alternate-day monitoring in acute phase; monthly for 6 months; then every 3 months
Steroid-intolerant patients; age <40 years; monocular patients; or bilateral cases with side effects from systemic corticosteroids	Immunosuppressants	Oral methotrexate 7.5–12.5 mg weekly	Daily in acute phase until healing; monthly for 6 months; then every 3 months
Bilateral cases with >3 quadrants affected, >50% stromal loss, impending perforation	High-dose systemic corticosteroids	Intravenous methylprednisolone; dose titrated based on clinical response	Inpatient care with internist collaboration; daily monitoring during acute phase; follow-up every 1–2 weeks for 3 months; then monthly for 3 months; every 3 months thereafter
Perforation or post-keratoplasty phase in severe bilateral cases	Combination immunosuppressive therapy	Intravenous methylprednisolone + Intravenous cyclophosphamide; dosage individualized per disease severity	Inpatient monitoring with multidisciplinary care; daily during acute phase; every 1–2 weeks for 3 months; monthly for 3 months; every 3 months thereafter
Refractory cases unresponsive to conventional immunosuppressants	Infliximab	Intravenous infusion at weeks 0, 2, and 6; monthly thereafter	Adhere to infusion schedule; hospital visits required for each dose and follow-up
Refractory cases unresponsive to conventional immunosuppressants	Adalimumab	Subcutaneous: 80 mg on days 0, 1, and 7; then 40 mg biweekly	Regular follow-up aligned with injection schedule
Refractory cases unresponsive to conventional immunosuppressants	Rituximab	Two Intravenous infusions of 1,000 mg each, 2 weeks apart	Follow infusion timeline; regular follow-up
Cases associated with systemic disease (e.g., Hepatitis C)	Interferon alpha-2b and ribavirin	Interferon alpha-2b 100 μg weekly; ribavirin 1,000 mg daily in divided doses (400 mg + 600 mg)	Monitor disease progression and liver function; regular follow-up; consult gastroenterologist if necessary

### 9.1 Medical treatment

Medical therapy follows a stepwise escalation: corticosteroids as first-line, conventional immunosuppressants as second-line, and biologics or monoclonal antibodies reserved for refractory disease.

#### 9.1.1 Corticosteroids

Topical corticosteroids (95), such as dexamethasone (55), betamethasone (38) and prednisolone (43), are commonly considered the initial treatment for MU, as same as other PUK (102). However, despite high-dose steroid therapy, there have been reported cases of progressive corneal melting (corneal lysis) in some patients [60].

#### 9.1.2 Immunosuppression

Immunosuppressive agents, such as cyclosporin A [CsA, topical 1% cyclosporin A eye drops, 2–4 times daily for 6–12 months (16) or systemic cyclosporin 10 mg/kg/day if necessary (103)], methotrexate (Table 6), cyclophosphamide (Table 6), tacrolimus [FK506, topically 0.02% tacrolimus ointment 1 to 3 times daily (104), azathioprine orally 2 mg/kg/day (103)] and mycophenolate mofetil [500 mg orally twice daily (105)], have demonstrated efficacy in treating MU in most studies and also considered another routine treatment for MU (9, 95, 103, 105–109). The choice between topical, oral, or intravenous immunosuppressive therapy should be tailored to the severity of the disease. However, systemic immunosuppressive therapy is related to the risk of secondary infections (109), underscoring its importance of rheumatologic or internal medicine guidance when administering such treatment.

All systemic immunosuppressive regimens require close internal-medicine supervision and regular hematologic and hepatic monitoring as summarized in Table 6 (101).

(a) Methotrexate is associated with gastrointestinal symptoms, cytopenia, and elevated liver enzymes. Warning signs such as fever, bruising, pallor, mouth ulcers, or respiratory complaints should prompt clinical attention, and patients should undergo biweekly liver function tests and blood counts during the first month, then monthly for six months, and subsequently every 2–3 months if stable.
(b) Cyclophosphamide may cause opportunistic infections, pneumonia, cystitis, mucositis, and cytopenia; thus, urinalysis, liver function tests, and complete blood counts should be conducted 10 days after the last dose and 2 days prior to the next dose.
(c) Cyclosporine and tacrolimus, both T-cell inhibitors, share common complications such as hypertension, cytopenia, and nephrotoxicity. Clinical signs like peripheral edema, mouth ulcers, rash, or elevated blood pressure warrant further evaluation. Monitoring includes blood counts every 2 days for the initial 6 weeks, then monthly, along with regular liver function tests and lipid profiles every 6 months.
(d) Azathioprine can lead to cytopenia, hepatotoxicity, and gastrointestinal disturbances; monthly liver function tests and blood counts are recommended for the initial 6–12 months, with reduced frequency to every 6–8 weeks upon stabilization.
(e) Mycophenolate mofetil may cause gastrointestinal symptoms, cytopenia, liver dysfunction, respiratory issues, or hematuria. Monitoring involves weekly blood counts during the first month and monthly assessments thereafter, including C-reactive protein, erythrocyte sedimentation rate, and liver function tests.

#### 9.1.3 Monoclonal antibody drugs

(a) Infliximab: Infliximab, a chimeric monoclonal antibody used for certain autoimmune diseases, is designed to bind TNF-*α* and prevent it from interacting with its target (110, 111). Two earlier reports from different European countries have simultaneously documented successful use of Infliximab in treating MU. Both studies suggest that Infliximab could be a significant option for preserving corneal when conventional immunosuppressive therapies fail (97, 98). Additionally, this therapy was recently applied in the treatment of two female MU patients, further substantiating its efficacy (96). Notably, all three reports emphasize that Infliximab was considered only after conventional immunosuppressants had proven ineffective. Infliximab may lead to congestive heart failure, multiple sclerosis, cutaneous vasculitis, injection site reactions, lymphoma, and opportunistic infections (101). Warning signs of these complications include pallor, bruising, chills, fever, and mouth ulcers. To ensure early detection, liver function tests and blood counts should be performed prior to every infusion, with annual dsDNA testing recommended.
(b) Adalimumab: The treatment mechanism of Adalimumab closely mirrors that of Infliximab, targeting TNF-*α* to inhibit its activity (110). Miguel et al. adopted this therapeutic approach for MU patients after conventional immunosuppressive treatments had failed. Their findings indicated that Adalimumab significantly alleviated the eye symptoms of MU patients and did not exhibit notable side effects during a 10-month period of long-term treatment (99). This suggests that Adalimumab, like Infliximab, could be a viable alternative for MU patients who do not respond to traditional immunosuppressive therapies. Adalimumab is associated with risks such as cytopenias, worsening or new onset of congestive heart failure, drug-induced lupus, lymphoma, opportunistic infections, and neurological disorders including multiple sclerosis (101). Alarming symptoms include pallor, bruising, chills, fever, and mouth ulcers. Monitoring protocols include monthly liver function tests and blood counts for the first three months, followed by testing every three months, with yearly dsDNA testing.
(c) Rituximab: Rituximab, as a chimeric monoclonal antibody against CD20 for treating certain autoimmune diseases and types of cancer, has emerged as a potential therapeutic option for MU (110). Guindolet et al. reported its effectiveness in managing severe cases of MU (100). Supriya et al. (112) emphasized its importance in controlling inflammation before surgery in MU patients. Rituximab can cause infusion reactions, opportunistic infections, cytopenia, and cardiovascular and dermatological complications (101). Pruritus, abdominal pain, chills, fever, dyspnea, pallor, and fatigue may signal the onset of adverse events. Monitoring includes vital sign assessment at each ophthalmic visit and regular blood counts.

#### 9.1.4 Interferon

Studies have shown that the combination of interferon alpha-2b and ribavirin is effective in treating MU in patients with hepatitis C virus infection (35, 36). The effectiveness of interferon in this context may stem from its immunomodulatory properties or from potential associations between MU and hepatitis C.

### 9.2 Surgical treatment

Surgical intervention is usually considered in the presence of severe corneal necrosis or the threat of perforation. It is advisable to proceed with surgery once inflammation is under control (113).

#### 9.2.1 Conjunctival resection

In cases where ulceration is attributed to autoimmunity, with the cornea serving as an antigen and the limbal conjunctiva containing antibodies and enzymes that contribute to corneal destruction, conjunctival resection may be considered. By limiting stromal antigen access to systemic circulation, conjunctival resection helps suppress immune infiltration and promotes ulcer repair. Consequently, perilimbal conjunctival excision has been proposed as a strategy to control inflammation. Brown et al. (114) performed conjunctival resection on 10 eyes diagnosed with MU, with only one eye experiencing ulcer recurrence post-surgery. However, Ikeda Lal et al. (115) argue that conjunctival resection fails to effectively halt disease progression or prevent recurrence. Controversy surrounds the efficacy of conjunctival resection due to limited case numbers and short follow-up periods. Larger studies are warranted to validate these clinical observations.

#### 9.2.2 Keratectomy

According to Brown et al. (114), keratectomy may be considered when medical treatments fail to resolve local corneal ulcers. This surgical intervention aims to remove the affected corneal tissue, thereby controlling corneal inflammation and preventing further development of corneal perforation.

#### 9.2.3 Amniotic membrane transplantation

Amniotic membrane (AM) transplantation facilitates epithelial cell adhesion and migration, induces epithelial differentiation, and suppresses interleukin secretion, thereby reducing corneal inflammation (116–118). Several studies have confirmed AM transplantation is effective in managing MU and other corneal ulcers unresponsive to medical therapy (117–119). Additionally, AM transplantation can be combined with other MU treatments. Lavaju P et al. (63) suggested that combining AM transplantation with autologous serum eye drops is an effective approach for MU. For MU patients at risk of or already experiencing corneal perforation, combining corneal transplantation with AM transplantation is feasible (120, 121). However, the effectiveness of AM transplantation for treating MU remains a topic of debate. Schallenberg et al. (122) concluded that while AM transplantation may not cure severe cases of MU, it can be beneficial in acute situations, such as critical corneal thinning, alongside immunosuppressive therapy.

#### 9.2.4 Lamellar keratoplasty

**Figure 4:**
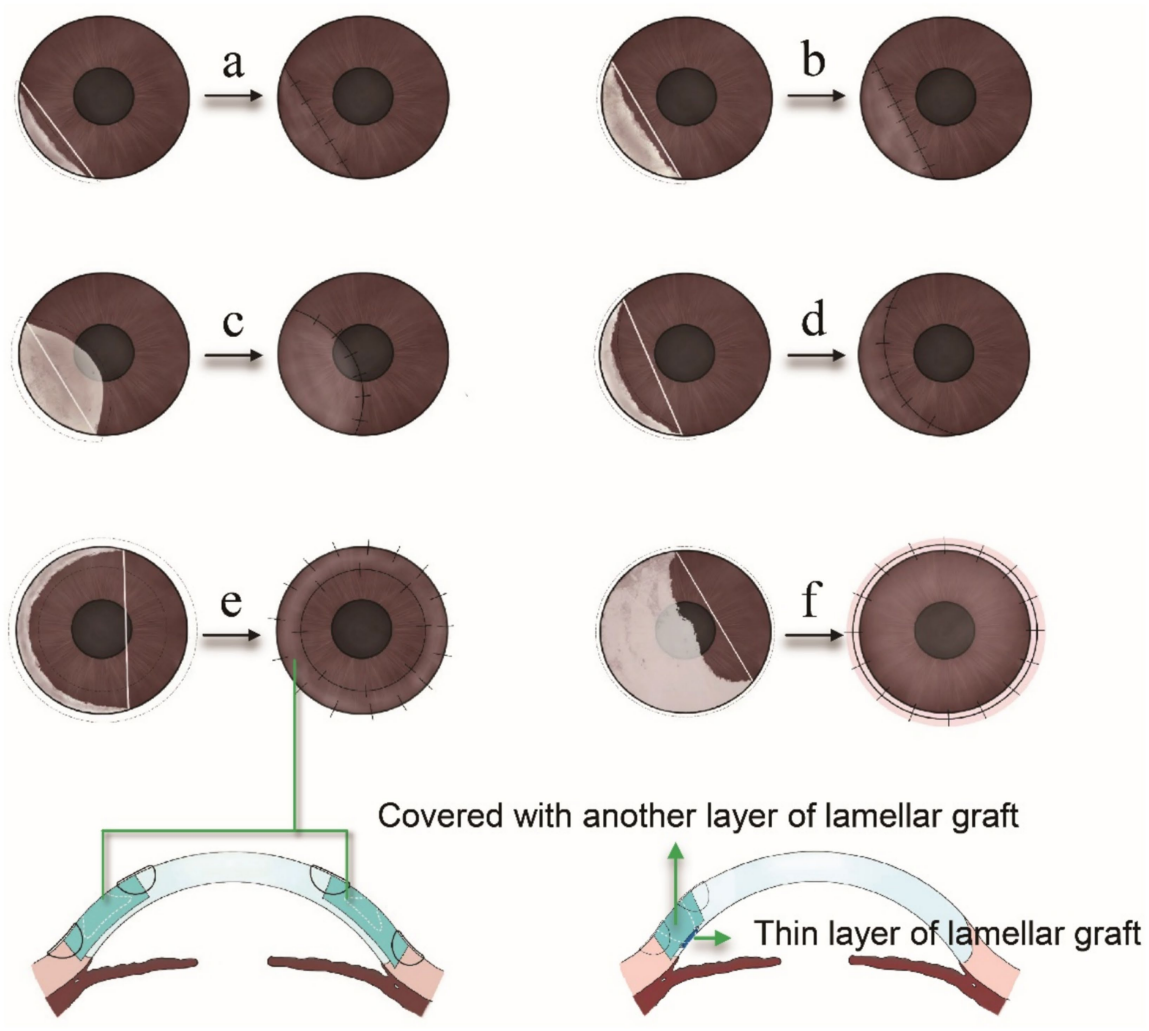
**(a)** When the ulcer involves less than 3 clock hours of the limbus and the chord connecting the lesion margins fully contains the corneal defect, a semilunar lamellar keratoplasty (LK) is performed. **(b)** For ulcers spanning 3 to 6 clock hours of the limbus, with the chord still encompassing the entire lesion, semilunar LK remains the preferred approach. **(c)** If the ulcer is less than 3 clock hours but involves part of the central cornea, biconvex LK is selected to ensure adequate excision. **(d)** When the ulcer spans 3 to 6 clock hours and the chord includes a significant portion of healthy cornea, crescentic LK is used to preserve uninvolved tissue within the chord. **(e)** For ulcers involving more than 6 clock hours without central corneal involvement, annular LK is employed to excise the peripheral lesion while preserving the central cornea. **(f)** If the ulcer exceeds 6 clock hours and extends into the central cornea, total LK is chosen to remove both peripheral and central lesions. **(g)** The corneal perforation or peripheral staphyloma formation, double-layer keratoplasty is chosen to repair the perforation and reconstruct the structure.

In addition to systemic immunosuppression, MU with severe corneal thinning or perforations often necessitates keratoplasty to remove the inflamed cornea and reconstruct its structure. However, penetrating keratoplasty (PK) frequently encounters challenges. Marta Jerez-Peña reported a case with persistent epithelial defects in the graft post-PK (113). Consequently, various forms of lamellar keratoplasty (LK) techniques have been proposed based on the extent and severity of corneal lesions:

(a) Semilunar/crescentic/biconvex LK: When the ulcer affects less than 6 clock hours of the limbus and does not involve the central cornea (Figures 4a,b), semilunar LK is the preferred technique. If the ulcer involves less than 3 clock hours but extends into the central cornea (Figure 4c), biconvex LK is selected to ensure adequate excision. When the ulcer spans 3 to 6 clock hours and the chord between both ends encompasses a substantial area of healthy cornea (Figure 4d), crescentic LK is chosen to preserve the uninvolved tissue.
(b) Annular LK: If the ulcer extends beyond 6 clock hours of the limbus without central corneal involvement (Figure 4e), annular LK is performed to remove the peripheral lesion while preserving the central cornea.
(c) Total LK: When the ulcer involves more than 6 clock hours and includes the central cornea (Figure 4f), total LK is required to excise both peripheral and central lesions. In such cases, large-diameter grafts (9–9.5 mm) are used, which carry a higher risk of immune rejection than standard-sized grafts. Therefore, enhanced local and systemic immunosuppressive therapy is necessary.
(d) Double-layer peripheral keratoplasty: Continued progression of MU can lead to corneal perforation and peripheral staphyloma formation. AM are too thin to repair corneal perforation and PK carries a higher risk of immune rejection. Therefore, double-layer keratoplasty has been proposed. The perforation is first repaired with a thin layer of lamellar graft incorporating the Descemet membrane, followed by an additional lamellar graft layer, the shape of which is determined by the ulcer’s configuration (Figure 4g). Shi et al. (123) performed double-layer peripheral keratoplasty in four patients with peripheral staphyloma (one of them with MU), all achieved favorable outcomes. It seems to be an ideal surgical intervention for MU with perforation or peripheral staphyloma.

Due to the complexity of MU, some recent reports have proposed non-traditional procedures that build upon PK or LK. Supriya et al. (112) combined PK with glaucoma valve surgery to achieve long-term visual recovery in MU patients. It should be noted, however, that such “customized surgical procedures (112)” are not standard and may even be a dangerous approach for other patients. Ashok et al. customized a new deep anterior lamellar keratoplasty for MU patients. They used trephines of two different diameters to achieve optimal donor sizing and avoid manual dissection (124). Notably, based on clinical experience, manual cutting of donor tissue is often challenging. Therefore, it is advisable to convert the shape of the ulcer into a geometric form, allowing for the use of two corneal trephines to harvest donor tissue of the desired shape and size. Additionally, a lamellar patch graft may be considered in cases involving both corneal melt and large corneal perforation.

In addition, Chen et al. (16) conducted a study involving 550 cases (715 eyes) of MU, where they combined LK with 1% cyclosporine A eye drops. They assert that LK represents an effective therapeutic approach for MU. However, some studies have reported higher recurrence rates associated with LK (125). It’s important to note that cases treated with LK are typically severe, and the observed increase in recurrence risk may not solely be attributed to the LK procedure.

#### 9.2.5 Boston type 1 keratoprosthesis

Boston type 1 keratoprosthesis (BKPro) represents a viable option for MU patients who have experienced failure with multiple corneal transplants (113). However, postoperative complications such as recurrence of MU, corneal necrosis, and uncontrolled high intraocular pressure might impact long-term treatment outcomes, necessitating prompt and intensive local and systemic interventions (126). While Sayan Basu et al. (127) reported promising short-term outcomes with BKPro in treating end-stage MU, the long-term efficacy of this approach requires further evaluation.

### 9.3 Other treatments

**Figure 5:**
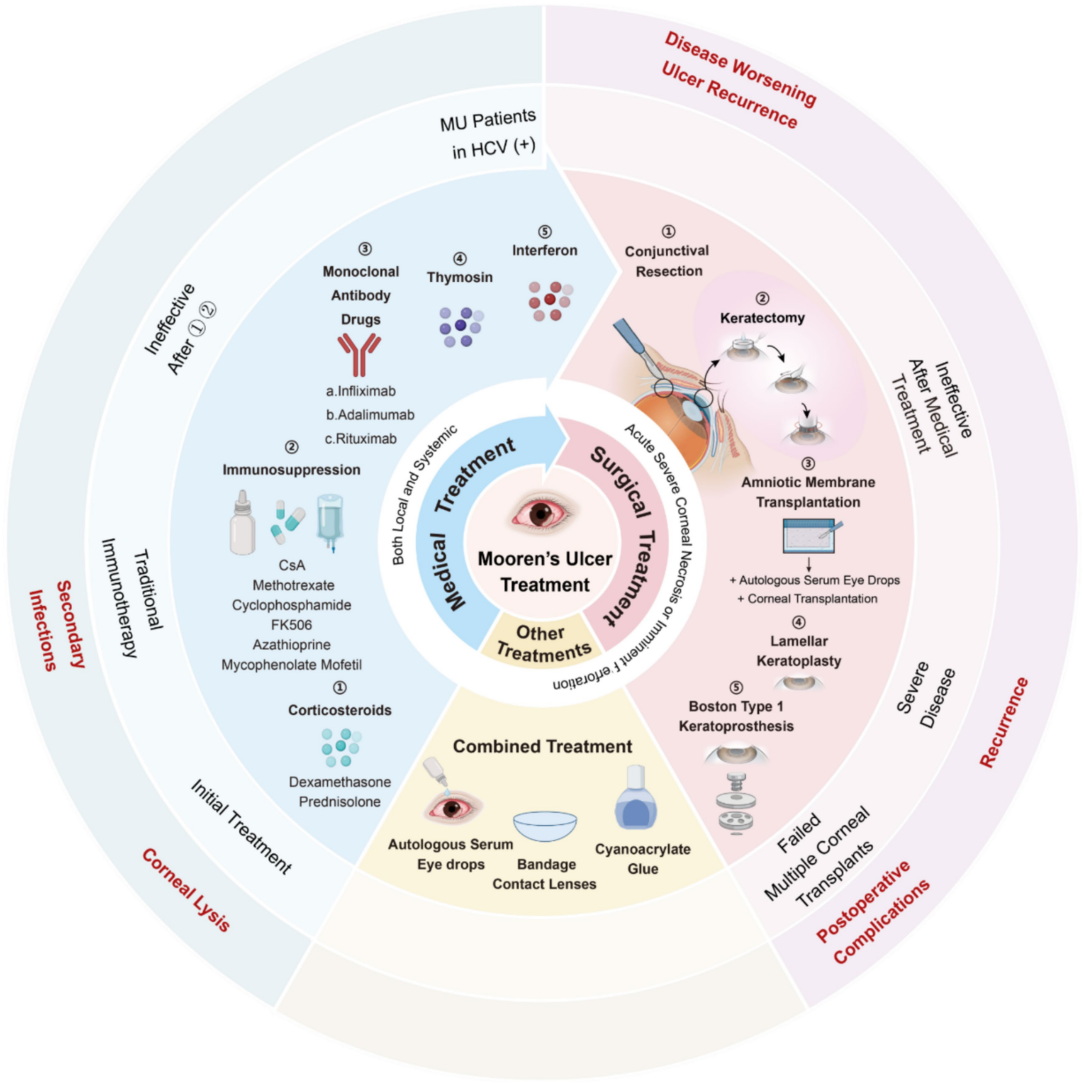
The treatment for Mooren’s ulcer. Created in BioRender. Wu, Y. (2025) https://BioRender.com/naj09b0.

Autologous serum, rich in nutrients and fibronectin, accelerates epithelial regeneration and repair. It contains α1 and α2 macroglobulins and the metalloproteinase inhibitor TIMP-1, which help prevent corneal ulcers and perforations. Consequently, many investigators have utilized autologous serum in MU patients to expedite corneal epithelial healing and prevent perforation (63, 128, 129). Bandage contact lenses are also recommended to alleviate patient discomfort, promote epithelial healing, and maintain corneal structural stability (115, 130). Immediate post-surgical use of bandage contact lenses can prevent damage to the Descemet’s membrane after conjunctival resection (131). Therefore, autologous serum eye drops and bandage contact lenses can be employed as a combined treatment approach for MU patients (Figure 5).

In addition, cyanoacrylate glue can be effectively used to repair corneal damage or perforations caused by MU. When MU leads to small, localized corneal ulcers, cyanoacrylate adhesive can be used for sealing, with a bandage contact lens subsequently applied to maintain corneal integrity (132). Notably, cyanoacrylate adhesive alone has been proven effective in managing corneal perforations ≤2.0 mm, providing prompt tectonic stabilization and preventing aqueous leakage (95). However, in MU patients with larger perforations (>2.0 mm), there is a significant risk that the adhesive may enter the anterior chamber and trigger severe intraocular inflammation. To mitigate this risk, a scleral patch graft can first be placed over the site of perforation (95), acting as a mechanical barrier to prevent intraocular extension of the glue. The scleral patch also serves as a biological scaffold for keratocytes, facilitating stromal regeneration and defect closure. In such cases, cyanoacrylate adhesive is subsequently applied over the patch to secure the repair (133). While these approaches have been shown to be effective in both the early and late stages of MU, they do not prevent the recurrence or ongoing progression of the disease (115).

## 10 Efficacy evaluation

The treatment of MU poses a considerable challenge, with systemic immunomodulatory therapy being pivotal for achieving successful functional and visual outcomes. Typically, changes in pain levels, photophobia, and inflammatory signs such as conjunctival hyperemia and edema are employed to assess alterations in the inflammatory response and treatment efficacy in MU patients (131). Shin Hatou et al. utilized IVCM to quantify ICD as a means of evaluating treatment response in MU patients (87). They observed that the mean ICD in patients with active MU was significantly higher than in remission, with effective treatment resulting in a gradual decrease in ICD over time. Limbal cysts detected during IVCM examination may serve as an indicator of impending perforation, necessitating close patient monitoring.

Recently, tear-based analysis has emerged as a promising approach in clinical and experimental practice (134). Given the localized immune hyperactivity implicated in MU pathogenesis, researchers have assessed corneal inflammatory status by analyzing levels of pro-inflammatory factors in MU patients’ tears (135, 136). This avenue holds potential as a novel method for evaluating treatment efficacy in MU patients. Future large-scale prospective studies are necessary to validate treatment outcomes, with careful consideration of disease severity, determined by age at onset, laterality, the extent (in clock hours) of corneal involvement, and stromal depth (4).

## 11 Prognosis

The progression of MU is closely intertwined with the healing process, involving neovascularization and epithelial regeneration. The process is thought to result from the coordinated proliferation and migration of endothelial progenitor cells and multipotent mesenchymal cells originating from the bone marrow (76, 88). Effective control of inflammation typically leads to ulcer healing, corneal vascularization, and subsequent corneal scar formation, resulting in reduced vision quality (137). However, if inflammation persists, it can culminate in corneal perforation.

The association between age and MU perforation remains a subject of debate. While Watson proposed a higher perforation rate in young men with BAM (12). But this is controversial in some studies. Muthaiah Srinivasan reported a perforation rate of 19% in 242 eyes of 166 patients, noting that it was not significantly more prevalent in bilateral cases or among young patients (138). Similarly, a study involving 715 eyes of 550 patients in China found no significant difference in perforation rates between young and old patients but observed a markedly higher rate in bilateral cases compared to unilateral ones (16). Kim (15) research revealed a significant association between younger age and corneal perforation, though whether the disease was bilateral or unilateral had no impact on perforation incidence. Given the rarity of MU, conducting large prospective studies on this disease is challenging. Consequently, further investigation into risk factors influencing the perforation rate is warranted. However, patients with MU who are younger or present with bilateral onset should be carefully monitored due to their potential for a higher perforation rate.

A recurrence of an epithelial defect associated with stromal infiltration is defined as a relapse in MU. Reports indicate a very high postoperative recurrence rate of 25.6%, with the first recurrence occurring mostly within 6 months after surgery. Recurrences may occur at the original lesion site or along the interface between the lamellar bed and the donor graft (16). Reducing the relapse rate is crucial for improving the cure rate of MU. Several factors have been associated with the early recurrence of MU. Yang et al. linked corneal infection and perforation to early recurrence (139), and Dong et al. identified male gender and severe cases requiring surgical treatment as risk factors (125). It is suggested that the mechanisms underlying recurrence may differ from those of the initial onset, necessitating further research to elucidate the risk factors, mechanisms, and optimal treatment strategies for MU recurrence.

Schallenberg et al. found disparities in the severity and prognosis of MU among different racial groups, proposing the expression of HLA-DR17 and/or HLA-DQ2 might influence the prognosis of MU (122). Although additional studies are needed to validate these findings, clinicians are advised to consider systemic immunosuppression for MU patients with high HLA-DR17 and/or HLA-DQ2 expression as early as possible.

## 12 Summary and future perspectives

The cornea, integral to the ocular surface ecosystem, collaborates with surrounding tissues to maintain ocular function and homeostasis (140–142). While minor corneal microenvironment abnormalities may go unnoticed due to compensatory mechanisms from neighboring tissues, the accumulation of severe dysfunction or abnormalities, as seen in conditions like MU, can trigger a cascade of complications. This may initiate a detrimental cycle of decompensation, exacerbating ocular pathology. MU has been recognized for over 170 years, yet its exact etiology, pathogenesis, and causal mechanisms remain unclear. Despite being identified as an autoimmune-related, genetically susceptible corneal disease leading to blindness, MU diagnosis remains one of exclusion, necessitating the exclusion of other causes of peripheral corneal ulcers (3, 20, 66). It is also one of the important reasons that may promote the process of MU in clinic. To address this, the work newly introduced Tables 2, 3 in this review after summarizing recent 19 case reports of MU, offering a structured summary of diagnostic examinations and an autoimmune serological panel, respectively. These tables aim to provide clinicians with practical guidance for differentiating MU from other causes of peripheral ulcerative keratitis, based on traditional diagnostic methods, and represent one of the novel contributions of this work.

In some new diagnostic technologies, although tools like 3D AS-OCT and IVCM have improved early detection, they have not overcome the core diagnostic challenge, reliably distinguishing MU from other causes of peripheral corneal ulceration. Similar inflammatory keratopathies may present comparable findings using these techniques, such as increased ICD and reduced visual quality in patients (143, 144). Furthermore, studies have indicated that these methods may not be as effective in examining the corneas of patients in MU remission compared to those with active corneal ulcers (87). Our previous work (56) introduced a histopathology-based diagnosis protocol that integrates AS-OCT and IVCM. While this protocol continues to rule out other similar diseases, it has proven effective in accurately diagnosing MU even when MU is obscured by other corneal conditions, such as pterygium. This protocol is a crucial tool for diagnosing MU in patients with complex ocular surface conditions. Future diagnostic criteria might include proteomic approach (52) and histopathological markers (51, 88, 89), like cathepsins, anti-citrullinated protein antibody (ACPA), catalase (CAT), CD34, CD74, KIT proto-oncogene, receptor tyrosine kinase (c-kit), G protein-coupled receptor 91 (GPR91), heat shock protein family A (Hsp70) member 5 (HSPA5), ICAM-1, leucine aminopeptidase 3 (LAP3), LFA-1, matrix metalloproteinase-10 (MMP-10), myocilin (MYOC), marginal zone B and B1 cell-specific protein (MZB1), NLR Family Pyrin Domain Containing 3 (NLRP3), peptidyl arginine deiminase 4 (PADI4), polymeric immunoglobulin receptor (PIGR), superoxide dismutase 2 (SOD2), superoxide dismutase 3 (SOD3), stromal cell surface marker 1 (STRO-1), and tissue inhibitor of metalloproteinase 3 (TIMP3), which could enhance the diagnostic accuracy and provide new insights into the underlying pathophysiology of MU.

In terms of treatment strategy, management of MU primarily aims to improve vision and corneal integrity, with varied approaches based on different subtypes’ clinical manifestations, prognosis, and treatment responses. Presently, similar to the treatment of most PUK, the majority of treatments focus on immune regulation, while surgical interventions prioritize lesion removal and corneal restoration (3).

Although MU can be managed with the intervention of advanced diagnostic protocols and treatment strategies aimed at restoring visual quality, the acute phase presentation and variable prognosis of MU patients pose significant challenges to the development of standardized treatment protocols. Notably, MU symptoms are significantly influenced by factors, including the patient’s race and age. Additionally, there are greater prognostic challenges associated with corneal perforation during exacerbations of MU, and there is a persistently high postoperative recurrence rate. Fortunately, recent advancements in diagnostic tools, such as 3D AS-OCT, IVCM, and proteomic markers, along with the use of immunosuppressants and monoclonal antibody therapies, have significantly improved the early detection and management of MU. These developments have enabled more effective disease control in many cases, potentially reducing the reliance on surgical interventions. However, despite these technological improvements, current diagnostic approaches still rely heavily on exclusion, and there remains no universally accepted clinical guideline or standardized treatment algorithm. Further research is needed to refine diagnostic criteria and establish evidence-based, consensus-driven treatment protocols.

## 13 Conclusion

Mooren’s ulcer (MU) is a rare but sight-threatening autoimmune corneal disease. Despite advances in immunopathology and imaging, its diagnosis still relies largely on exclusion, and management remains challenging. Early recognition and prompt initiation of immunosuppressive therapy are crucial to prevent corneal perforation and vision loss. A tiered, individualized “stepladder” approach, beginning with corticosteroids and escalating to systemic immunomodulators, biologics, or surgery as required, offers the most effective management strategy. For clinicians, establishing a structured diagnostic workup and close interdisciplinary collaboration between ophthalmologists and immunologists is essential. For researchers, future efforts should focus on defining standardized diagnostic criteria, identifying reliable immunologic biomarkers, and validating evidence-based treatment algorithms through multicenter studies.

## 14 Methods of literature search

The search of this comprehensive review was performed in the PubMed and Web of Science databases (up to Oct 2025), without limitations on publication date or type. Articles not published in English or lacking peer review were excluded. This narrative review incorporated a range of relevant keywords and phrases, including but not limited to: “Mooren,” “peripheral ulcerative keratitis,” or “Mooren’s ulcer.” All articles deemed pertinent to the study, as determined by the two authors’ discretion, were included in the review.

## Glossary

3D AS-OCT: Three-dimensional anterior segment optical coherence tomography
ACPA: Anti-citrullinated protein antibody
AM: Amniotic membrane
BAM: Bilateral aggressive mooren’s ulceration
BCVA: Best-corrected visual acuity
BIM: Bilateral indolent mooren’s ulceration
BKPro: Boston type 1 keratoprosthesis
C1: Complement 1
CAGC: Calcium granule protein C
CAT: Catalase
CICs: Circulating immune complexes
c-kit: KIT proto-oncogene, receptor tyrosine kinase
CO-Ag: Cornea-associated antigen
CsA: Cyclosporin A
CVDs: Collagen vascular diseases
GPA: Granulomatosis with polyangiitis
GPR91: G protein-coupled receptor 91
HCV: Hepatitis c virus
HE: Hematoxylin–eosin
HLA: Human leukocyte antigen
HSPA5: Heat shock protein family A (Hsp70) member 5
ICAM-1: Intercellular adhesion molecule-1
ICD: Inflammatory cell density
IHC: Immunohistochemical
IVCM: *In vivo* confocal microscopy
LAP3: Leucine aminopeptidase 3
LFA-1: Lymphocyte function-associated antigen-1
LK: Lamellar keratoplasty
MMP-10: Matrix metalloproteinase-10
MU: Mooren’s ulcer
MYOC: Myocilin
MZB1: Marginal zone B and B1 cell-specific protein
NLRP3: NLR family pyrin domain containing 3
PADI4: Peptidyl arginine deiminase 4
PIGR: Polymeric immunoglobulin receptor
PK: Penetrating keratoplast
PMD: Pellucid marginal degeneration
PUK: Peripheral ulcerative keratitis
SOD2: Superoxide dismutase 2
SOD3: Superoxide dismutase 3
STRO-1: Stromal cell surface marker 1
Th: Helper T cells
TIMP3: Tissue inhibitor of metalloproteinase 3
TMD: Terrien’s marginal degeneration
Ts: T cells
UM: Unilateral Mooren’s ulceration
